# Discoidin domain receptor 1 is a potential target correlated with tumor invasion and immune infiltration in gastric cancer

**DOI:** 10.3389/fimmu.2022.933165

**Published:** 2022-07-22

**Authors:** Songna Wang, Yuan Fu, Kudelaidi Kuerban, Jiayang Liu, Xuan Huang, Danjie Pan, Huaning Chen, Yizhun Zhu, Li Ye

**Affiliations:** ^1^ Minhang Hospital and Department of Biological Medicines at School of Pharmacy, Fudan University, Shanghai, China; ^2^ Shanghai Engineering Research Center of Immunotherapeutics, School of Pharmacy, Fudan University, Shanghai, China; ^3^ School of Pharmacy, Macau University of Science and Technology, Macao, Macao SAR, China

**Keywords:** DDR1, gastric cancer, invasion, prognosis, immune infiltration

## Abstract

Discoidin domain receptor 1 (DDR1) has been demonstrated to be able to promote tumor invasion and metastasis and being closely related to tumor immune infiltration. However, DDR1 has rarely been studied in gastric cancer. Here, we primarily evaluated DDR1 expression in gastric cancer and its cell lines using multiple databases. Subsequently, the cancer prognosis was investigated in relation to DDR1 expression. After analysis, we discovered that DDR1 was highly expressed and significantly connected with poor prognosis in gastric cancer. To comprehensively understand the molecular mechanism of DDR1, we explored genes and proteins interacting with DDR1 in gastric cancer using databases. Additionally, we found that the expression level of DDR1 was inversely correlated with immune infiltration and significantly relative to various immune cell markers. Overall, DDR1 was implicated in invasion, metastasis, and immune infiltration of gastric cancer. Inhibition of DDR1 may have the potential to alleviate the strong invasiveness and metastasis of advanced gastric cancer. Meanwhile, immune exclusion by DDR1 may also provide a new strategy for improving the efficacy of immune checkpoints inhibitors (ICIs), such as programmed cell death protein 1 (PD-1) antibody.

## Introduction

Globally, gastric cancer, also called stomach cancer, is the third leading cause of death from cancer (1). One of the most important reasons for its poor prognosis is that it is usually diagnosed at an advanced stage (2), which is characterized by strong invasion and metastasis (3). Although gastric cancer is treated primarily by surgery, the efficacy of surgery is low for patients in advanced stage (4). During the past few years, immune checkpoints inhibitors (ICIs) have been shown to be effective against several solid tumors, but have had limited approval in gastrointestinal cancers (5). Clinical data showed that patients treated with programmed cell death protein 1 (PD-1) antibodies had low response rate in advanced gastric cancer (6). It has been found that ICIs’ effectiveness requires the presence of a strong immune infiltration and the ability to produce an effective antitumor response (7). Moreover, infiltration of immune cells is particularly associated with patient prognosis (8). Therefore, there is an urgent need to improve patient sensitivity to ICIs through exploring potential regulatory mechanisms of immune cell infiltration.

DDR1 is a type of collagen receptor with tyrosine kinase activity, which has five isoforms through alternative splicing (9). Cell adhesion, migration, proliferation, and extracellular matrix (ECM) remodeling are regulated by its interactions with ECM components (10, 11). Mounting evidence shows that DDR1 expression is significantly upregulated in a variety of cancers, including ovary, breast, colon, and lung cancers (12–14). It is also associated with malignant behaviors of tumors, such as tumor cells proliferation, invasion, and metastasis (15). Research indicated that the cross-talk between DDR1 and signal transducer and activator of transcription 3 (STAT3) promoted the progression of hepatocellular carcinoma (HCC) *via* epithelial–mesenchymal transition (EMT) and glutamine metabolism (16). In pancreatic cancer, collagen stimulated CXC chemokine ligand-5 (CXCL5) production through the DDR1/PKCθ/spleen tyrosine kinase (SYK)/nuclear factor κB (NF-κB) pathway, which induced neutrophil extracellular traps (NETs) to drive tumor metastasis (17). Other studies also reported that DDR1 could increase invasion and metastatic spread of gastric cancer *via* EMT (18). Research indicated that increased apoptosis and decreased migration in breast cancer were observed when patients were treated with DDR1 inhibitor nilotinib (19). These suggests that inhibition of DDR1 may be beneficial for the treatment of advanced gastric cancer.

Notably, recent studies have found that DDR1 can control certain properties of immune cells. The molecular structure of DDR1 consists of three major domains, including a transmembrane domain, an intracellular kinase domain, and an extracellular domain (ECD) (20). One study proposed that the ECD of DDR1, rather than its intracellular kinase domain, enhanced the alignment of collagen fibers and blocked immune infiltration by binding to collagen (21). Similarly, it was reported that DDR1 expression exhibited inverse correlation with intratumoral T-cell abundance in triple-negative breast cancer (22). Anti-DDR1-ECD monoclonal antibody resulted in fewer and shorter arrangements of collagen fibers at the tumor edge, which enhanced immune cell infiltration, increased the total number of infiltrating CD8^+^ and CD4^+^ T cells, and promoted interferon gamma (IFN-γ) production (21). This mechanism of immune exclusion provides a new strategy for improving the effectiveness of ICIs such as PD-1 antibody. Drugs targeting DDR1-ECD will help to reduce immune exclusion in gastric cancer, enhance T-cell infiltration, and reduce NETs, thereby improving the tumor immune microenvironment and slowing tumor progression (23).

Herein, we used a variety of databases including Tumor Immunoassay Resource (TIMER), UCSC Xena, Gene Expression Display Server (GEDS), UALCAN, Gene Expression Profiling Interactive Analysis (GEPIA), Kaplan–Meier plotter, and PrognoScan to study DDR1 gene expression and its impact on prognosis in multiple cancers. We found that DDR1 expression levels were highly upregulated in many cancers, and highly expressed DDR1 significantly affected the prognosis of gastric cancer. Subsequently, genes and proteins interacting with DDR1 were analyzed through STRING, PINA, and Metascape databases. Finally, the effect of DDR1 expression in gastric cancer on immune cell infiltration was investigated through TIMER database. Results indicated that DDR1 expression was negatively related to the infiltration of various immune cells, especially macrophages. Thus, as described in our study, DDR1 might be a potential target for gastric cancer. In addition, it provides a new approach for improving ICI therapy efficacy by enhancing immune infiltration.

## Materials and methods

### Expression analysis of DDR1

Through TIMER database (https://cistrome.shinyapps.io/timer/), DDR1 expression levels in various cancers were analyzed (24, 25). Test of Wilcoxon significance was performed for differential expression. Subsequently, we used the UCSC Xena online platform (https://xena.ucsc.edu) to assess differences of DDR1 expression between stomach adenocarcinoma (STAD) and normal tissues (26). We obtained gene expression data of 544 STAD patients. Differential expression analysis was performed using Welch’s test. By using GEDS (http://bioinfo.life.hust.edu.cn/web/GEDS/), we examined DDR1 expressions in 37 gastric cancer cell lines (27).

### UALCAN

UALCAN database (http://ualcan.path.uab.edu/index.html) performed the multifaceted analysis about DDR1 expression in STAD using data from The Cancer Genome Atlas (TCGA) (28). The contents of the analysis include sample types, patient’s gender, TP53 mutation status, individual cancer stages, nodal metastasis status, tumor grades, patient’s age, and histological subtypes. p-value < 0.05 was considered statistically significant.

### PrognoScan database analysis

By searching vast cancer microarray datasets that are publicly available, PrognoScan database (http://dna00.bio.kyutech.ac.jp/PrognoScan/) can effectively help to explore the influences of various gene expression on patients’ prognosis, thus evaluating potential markers and targets in oncotherapy (29). We first explored the relationship between DDR1 levels and survival situation in different cancers *via* PrognoScan database. When the p-value was <0.05, it indicated that there was a significant correlation between DDR1 levels and the prognosis of each tumor type and subtype.

### Kaplan–Meier plotter

Based on gene chips and RNA-seq data from public databases such as Gene Expression Omnibus (GEO), European Genome–Phenome Archive (EGA), and TCGA, the Kaplan–Meier plotter (http://kmplot.com/analysis/) provides the correlation analysis between a variety of gene expressions and prognosis in 21 cancer types (30). To investigate the prognostic impact of DDR1 expression level, we first used the Kaplan–Meier plotter in breast, ovarian, lung, and gastric cancers because their cohorts possess relatively large sample sizes. It is worth noting that those patient samples were grouped by an automatically selected best cutoff for optimal performance. Moreover, it was further employed to investigate the influences of various clinicopathological characteristics in gastric cancers.

### GEPIA

Based on a mass of RNA-sequencing expression data, GEPIA (http://gepia.cancer-pku.cn/index.html) offers a powerful platform to conduct genetic analysis (31). In the “single gene analysis” module of GEPIA, we first generated prognosis curves in 33 divergent types and subtypes of cancers, with DDR1 expressing differently. Additionally, GEPIA was also employed to analyze the links between DDR1 and the specific markers of divergent tumor-infiltrating immune cells (TIICs). The analysis was performed using tumor and normal tissue datasets.

### Analysis of genes and proteins that interact with DDR1

Using STRING (https://string-db.org/) (version 11.5), we constructed a protein–protein interaction (PPI) network for DDR1 and related proteins (32–34). The statistical significance of an interaction was established when the combined score was > 0.4. Subsequently, the interaction network was further analyzed and visualized using Cytoscape (version 3.8.2). We also further analyzed genes and proteins interacting with DDR1 in STAD using the PINA database (https://omics.bjcancer.org/pina/) (version 3.0) (35–37).

### Tumor-infiltrating immune cells analysis

We further investigated the influences of DDR1 expression level on the infiltration levels of specific immune cell subsets in STAD and lymphoid neoplasm diffuse large B-cell lymphoma (DLBC) using TIMER database. Then, we visualized the survival differences for immune infiltration correlated to DDR1 in STAD and DLBC. Simultaneously, the correlation between DDR1 expression and different immunomarker sets was explored *via* TIMER database. Partial Spearman’s correlation adjusted by purity was applied to assess their relationships.

### Datasets

All datasets used in this study are publicly available, but there are certain differences in different databases. Among them, datasets used by TIMER, UCSC Xena, UALCAN,and GEPIA are mainly based on STAD-TCGA and DLBC-TCGA. In addition, datasets used by GEDS come from Cancer Cell Line Encyclopedia (CCLE); datasets used in PrognoScan analysis have been marked in the figures, including GSE12417-GPL96, GSE7696, GSE26712, jacob-00182-HLM, GSE16560, GSE2658, E-TABM-158, and GSE17536; and datasets used by Kaplan–Meier plotter are the expression data of six mRNA chips in GEO, including GSE14210, GSE15459, GSE22377, GSE29272, GSE51105, and GSE62254.pt?>

## Results

### DDR1 is highly expressed in gastric cancer

**Figure 1:**
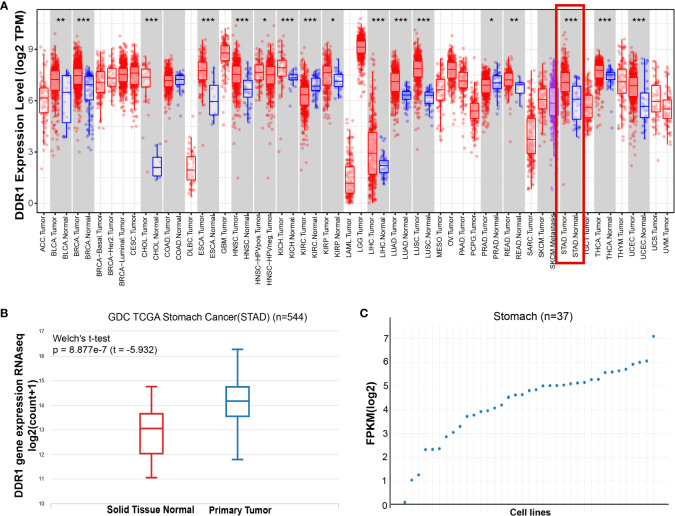
The expression level of DDR1 in gastric cancer. **(A)** DDR1 expression levels in multiple tumors and normal tissues were analyzed using the TIMER database (*p < 0.05, **p < 0.01, ***p < 0.001). **(B)** The mRNA level of DDR1 was examined in STAD using UCSC Xena database. Welch’s t-test was used for statistical difference. **(C)** A scatter plot of DDR1 expressions in 37 gastric cancer cell lines using the GEDS platform.

We first assessed DDR1 expression in multiple tumors and normal tissues by the TIMER database. In comparison with normal tissues, the expression levels of DDR1 were significantly higher in bladder urothelial carcinoma (BLCA), breast invasive carcinoma (BRCA), cholangiocarcinoma (CHOL), esophageal carcinoma (ESCA), head and neck squamous cell carcinoma (HNSC), kidney chromophobe (KICH), kidney renal clear cell carcinoma (KIRC), kidney renal papillary cell carcinoma (KIRP), liver hepatocellular carcinoma (LIHC), lung adenocarcinoma (LUAD), lung squamous cell carcinoma (LUSC), prostate adenocarcinoma (PRAD), rectum adenocarcinoma (READ), STAD, thyroid carcinoma (THCA), and uterine corpus endometrial carcinoma (UCEC) (**Figure 1A**). Consistently, the analysis result of UCSC Xena also confirmed that the expression of DDR1 was markedly elevated in STAD (**Figure 1B**). Furthermore, we interrogated DDR1 expression in 37 gastric cancer cell lines using the GEDS platform (**Figure 1C**; **Supplementary Table S1**). Of these, NCC-StC-K140 cells show the highest DDR1 expression, while SNU-1 cells show the lowest. They can be used to study the effect of DDR1 expression on gastric cancer.

### DDR1 expression levels in the context of different clinical parameters of gastric cancer

**Figure 2:**
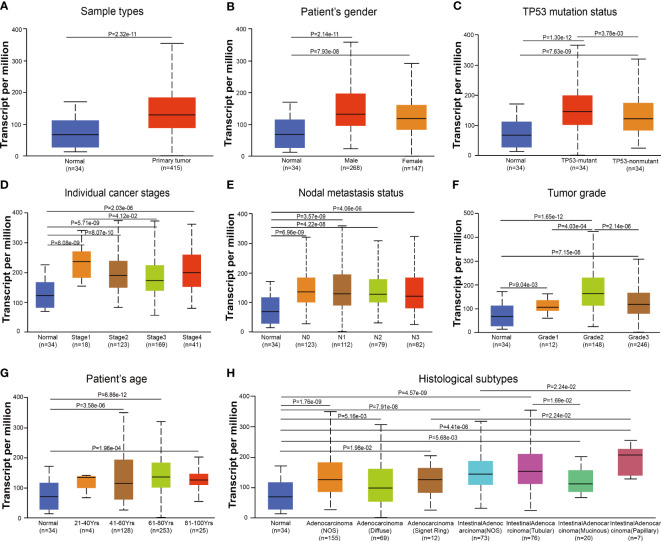
DDR1 expression is evaluated in diverse stages based on clinical characteristics by UALCAN database. Analysis of DDR1 expression based on sample types **(A)**, gender **(B)**, TP53 mutation status **(C)**, individual cancer stages **(D)**, nodal metastasis status **(E)**, tumor grades **(F)**, age **(G)**, and histological subtypes **(H)**. Marking the central point is the median. [N0, no regional lymph node metastasis; N1, metastases in one to three axillary lymph nodes; N2, metastases in four to nine axillary lymph nodes; N3, metastases in 10 or more axillary lymph nodes; Grade 1, well differentiated (low grade); Grade 2, moderately differentiated (intermediate grade); Grade 3, poorly differentiated (high grade); Grade 4, undifferentiated (high grade)].

Based on samples from TCGA-STAD in the UALCAN database, DDR1 expression levels in the context of various clinical parameters of gastric cancer were examined. Gastric cancer exhibited significantly increased DDR1 expression compared to normal tissues (**Figure 2A**). In a similar vein, this trend was observed in both male and female patients (**Figure 2B**). In contrast with mutant TP53, the non-mutant TP53 showed significantly lower DDR1 expression (**Figure 2C**). On the basis of individual cancer stages, DDR1 expressions of STAD were markedly higher in stages 1–4 (**Figure 2D**). Similarly, this increase was observed in N0, N1, and N2 stages (**Figure 2E**). Considering the tumor grades, DDR1 was more expressed in tumor grades 1–3 than in normal control (**Figure 2F**). Moreover, DDR1 expression of grade 2 was significantly higher than those of grades 1 and 3. With respect to age, DDR1 expression increased significantly in patients over 40 years of age (**Figure 2G**). Additionally, high DDR1 expressions were observed in various histological subtypes of STAD (**Figure 2H**).

### DDR1 expression correlates with prognosis of cancer patients

**Figure 3:**
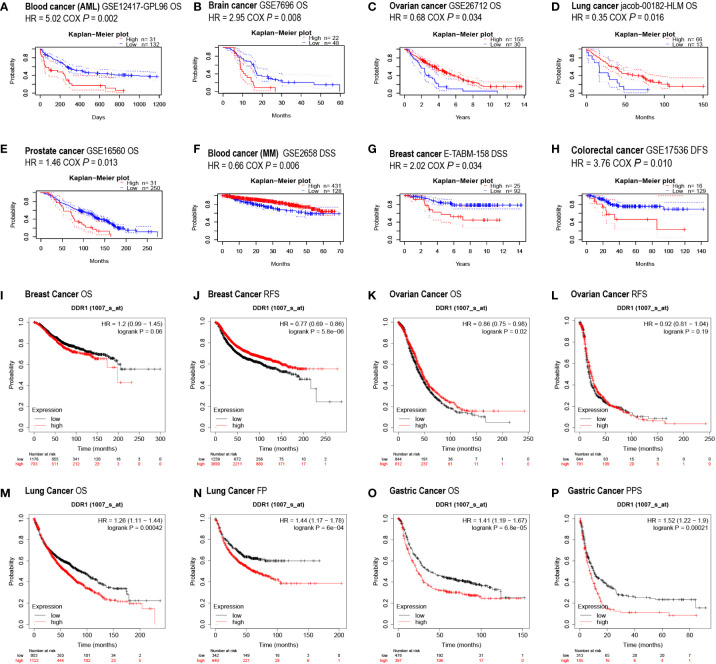
Effect of DDR1 expression on different cancers prognosis using PrognoScan and Kaplan–Meier plotter databases. **(A–H)** Survival curves were assessed using PrognoScan database. Survival curves of OS in blood cancer (AML) **(A)**, brain cancer **(B)**, ovarian cancer **(C)**, lung cancer **(D)**, and prostate cancer **(E)** are shown. Survival curves of DSS in blood cancer (MM) **(F)** and breast cancer **(G)** are showed. Survival curve of DFS in the colorectal cancer **(H)** is shown. **(I–P)** Survival curves were analyzed using Kaplan–Meier plotter database. Survival curves of OS **(I)** and RFS **(J)** in breast cancer, OS **(K)** and RFS **(L)** in ovarian cancer, OS **(M)** and FP **(N)** in lung cancer, and OS **(O)** and PPS **(P)** in gastric cancer. AML, acute myelogenous leukemia; MM, multiple myeloma; OS, overall survival; DSS, disease-specific survival; DFS, disease-free survival; RFS, relapse-free survival; FP, first progression; PPS, post-progression survival.

To investigate the prognostic value of DDR1 as a target for cancer patients, the PrognoScan database was first employed to evaluate the effect of different DDR1 expression levels on survival situation in patients with multiple cancer types. Preliminary results indicated that the expression of DDR1 was significantly related to the prognosis of patients with various cancer types, including blood, brain, ovarian, lung, prostate, breast and colorectal cancers (**Figures 3A–H**). Interestingly, with regard to different cancer types and even subtypes, DDR1 expression may be inversely correlated to prognosis. For example, elevated DDR1 expression was significantly correlated with poorer prognosis in acute myelogenous leukemia (AML) but better prognosis in multiple myeloma (MM) (**Figures 3A, F**).

In addition, the Kaplan–Meier plotter database was also applied to evaluate the prognostic relevance of DDR1 expression levels in various cancers. The elevation of DDR1 expression was observed to be significantly correlated to poor prognosis in patients with lung cancer (OS HR = 1.26, p = 0.00042; FP HR = 1.44, p = 6e−04) and gastric cancer (OS HR = 1.41, p = 6.8e−05; PPS HR = 1.52, p = 0.00021) (**Figures 3M–P**). However, there was not such a concordant and significant association between the DDR1 expression and the prognosis in breast and ovarian cancer patients (**Figures 3I–L**).

Eventually, we went the extra mile to investigate the survival curves of 33 TCGA cancer types using GEPIA database, revealing DDR1 expression to be significantly correlated with DFS in CHOL and KICH, OS in mesothelioma (MESO), and both OS and DFS in KIRC (**Supplementary Figures S1**–**3**). According to the above analysis, DDR1 expression is clearly demonstrated to be significantly correlated to poorer prognosis across diverse cancer types.

### Confirmation of the prognostic value of DDR1 with various clinicopathological characteristics of gastric cancer

Since we have noticed the significant impact of DDR1 expression on the prognosis of gastric cancer patients, the prognostic value of DDR1 was further evaluated according to various clinicopathological characteristics of gastric cancer by virtue of the Kaplan–Meier plotter database (**Table 1**). It turned out that the high expression of DDR1 closely related to poor prognosis of both female (OS HR = 1.74, p = 0.002) and male (OS HR = 1.29, p = 0.021; PPS HR = 1.49, p = 0.0026) gastric cancer patients. Specifically, overexpression of DDR1 was significantly correlated with worse OS and PPS in stage 1 (OS HR = 3.21, p = 0.022; PPS HR = 10.27, p = 0.0077) and stage 3 (OS HR = 1.7, p = 0.00026; PPS HR = 2.07, p = 0.00096). In the four N categories and two M categories, stage N2 (OS HR = 2.12, p = 0.0011; PPS HR = 2.28, p = 0.00085) and stage M1 (OS HR = 2.43, p = 0.005; PPS HR = 3.27, p = 0.0025) had the highest HR values of both OS and PPS. Taken together, these results suggest that the high expression of DDR1 affects the prognosis of different gastric cancer classifications to varying degrees.

**Table 1:** Correlation between DDR1 expression and different clinicopathological characteristics in gastric cancer *via* Kaplan–Meier plotter.

Clinicopathological characteristics	OS (n = 881)			PPS (n = 503)		
	N	HR (95%CI)	p-value	N	HR (95%CI)	p-value
**Sex**						
** Female**	236	1.74 (1.22–2.48)	**0.0020**	149	1.53 (0.98–2.38)	0.060
** Male**	544	1.29 (1.04–1.60)	**0.021**	348	1.49 (1.15–1.93)	**0.0026**
**Stage**						
** 1**	67	3.21 (1.11–9.28)	**0.022**	31	10.27 (1.23–85.58)	**0.0077**
** 2**	140	0.76 (0.36–1.58)	0.45	105	0.63 (0.32–1.22)	0.17
** 3**	305	1.70 (1.28–2.27)	**0.00026**	142	2.07 (1.33–3.22)	**0.00096**
** 4**	148	0.71 (0.47–1.08)	0.11	104	0.73 (0.44–1.20)	0.21
**Stage T**						
** 2**	241	0.61 (0.40–0.93)	**0.020**	196	0.66 (0.42–1.04)	0.069
** 3**	204	1.77 (1.25–2.52)	**0.0013**	150	1.98 (1.32–2.95)	**0.00071**
** 4**	38	0.29 (0.12–0.70)	**0.0037**	29	0.38 (0.12–1.15)	0.077
S**tage N**						
** 0**	74	0.59 (0.25–1.40)	0.23	41	0.24 (0.06–1.02)	**0.037**
** 1**	225	1.31 (0.86–2.00)	0.21	169	1.55 (0.95–2.54)	0.079
** 2**	121	2.12 (1.33–3.35)	**0.0011**	105	2.28 (1.39–3.75)	**0.00085**
** 3**	76	0.51 (0.29–0.90)	**0.019**	63	0.57 (0.31–1.03)	0.061
** 1 + 2 + 3**	422	0.83 (0.64–1.09)	0.18	337	1.3 0(0.97–1.76)	0.081
**Stage M**						
** 0**	444	0.85 (0.64–1.13)	0.25	342	1.38 (1.01–1.88)	**0.04**
** 1**	56	2.43 (1.28–4.61)	**0.0050**	36	3.27 (1.46–7.32)	**0.0025**
**Lauren classificatio**n						
** Intestinal**	320	1.86 (1.34–2.59)	**0.00018**	192	2.51 (1.65–3.83)	**0.0000098**
** Diffuse**	241	0.78 (0.55–1.11)	0.17	176	0.81 (0.55–1.20)	0.29
**Differentiation**						
** Poor**	165	1.44 (0.89–2.31)	0.13	49	0.60 (0.31–1.18)	0.13
** Moderate**	67	1.64 (0.81–3.31)	0.16	24	1.8 0(0.59–5.49)	0.29

p-value of log-rank test compares survival curves between patients with high DDR1 expression and those with low DDR1 expression. Bold values indicate p-value <0.05. OS, overall survival; PPS, post-progression survival; HR, hazard ratio.

### Genes and proteins that interact with DDR1 in gastric cancer

**Figure 4:**
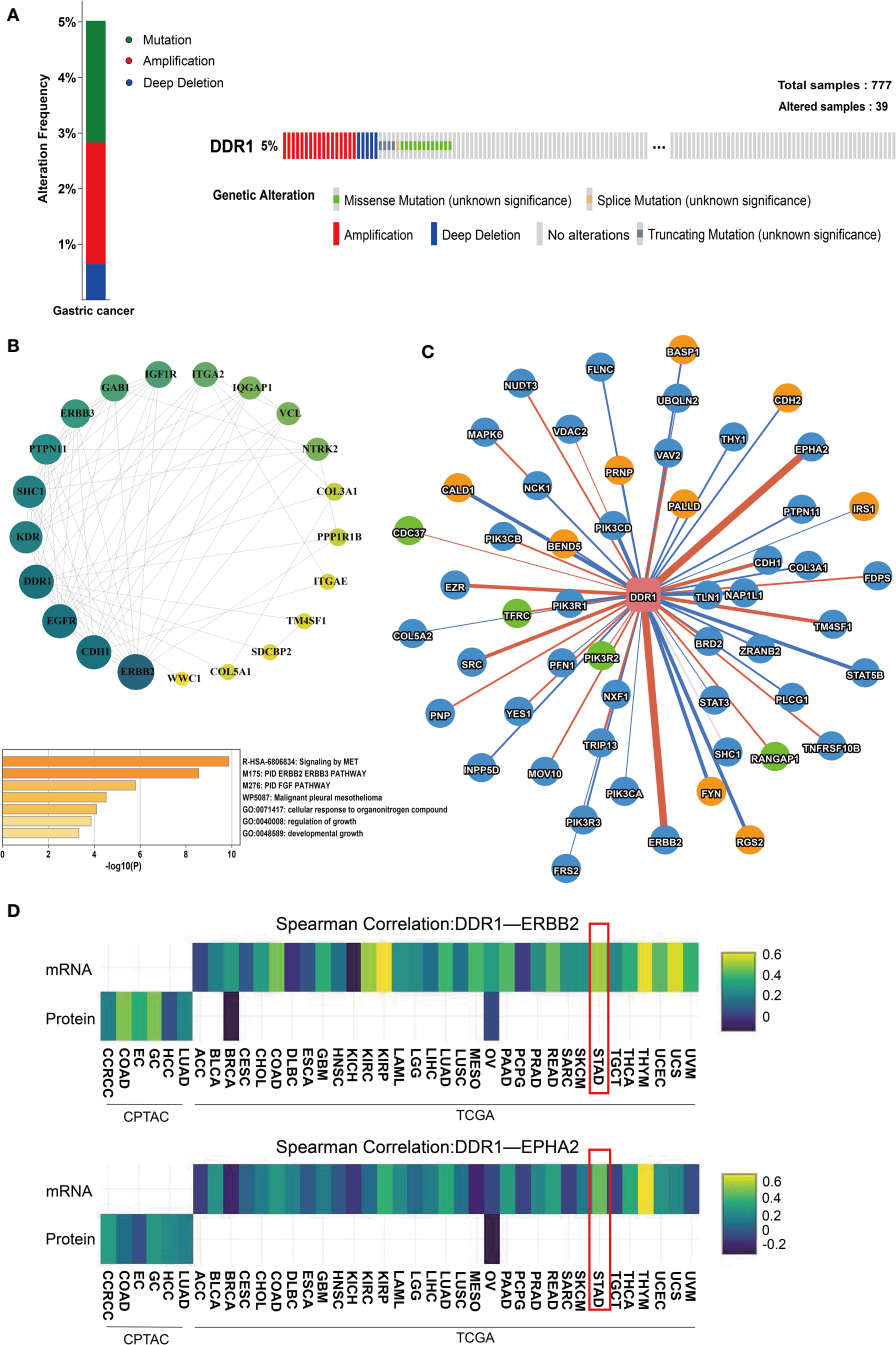
Analysis of genes and proteins that interact with DDR1. **(A)** The OncoPrint of DDR1 gene alterations in queried 777 samples of gastric cancer using cBioPortal database. Colors are used to highlight the various genetic alterations. **(B)** A PPI network of DDR1 and its related proteins using the Cytospace software and heatmaps of pathway enrichment using the Metascape. The p-value cutoff is 0.01. **(C)** The interaction network of genes and proteins that interact with DDR1 using PINA database. The yellow nodes represent genes related to poor prognosis (p <0.05, HR>1). The green nodes represent genes connected with good prognosis (p <0.05, HR<1). The red edges represent a positive correlation (FDR <0.05), while the blue edges represent a negative correlation (FDR <0.05). Edge width is relative to correlation coefficients. **(D)** Heatmap shows the correlation coefficients of mRNA expression (top row) and protein abundance (bottom row) among interacting proteins (DDR1-ERBB2 and DDR1-EPHA2) in each tumor type.

First, we analyzed DDR1 mutations in gastric cancer using the cBioPortal database. Out of 777 samples, DDR1 gene was altered in 39 (5%) samples (**Figure 4A**). Most of these mutation types were amplification and deep deletion. There were also a small number of missense mutation, splice mutation, and truncating mutation that might result in unfunctional DDR1. To learn more about the molecular mechanisms involved in DDR1, we used STRING website to search the relationship between DDR1 and its related proteins, while the Cytospace software was applied to generate the network map (**Figure 4B**). DDR1 was closely related to SHC1 (combined score, 0.949), PTPN11 (combined score, 0.941), TM4SF1 (combined score, 0.904), and WWC1 (combined score, 0.846). Whereafter, we used Metascape database for pathway enrichment analysis for the genes above. Pathways were mainly enriched in MET signaling and PID ERBB2 ERBB3 pathway. Considering that the mechanisms of action inside genes differ in different diseases, we used the PINA database to further analyze genes and proteins that interact with DDR1 in STAD. The interaction network diagram of the interacting genes with DDR1 in STAD is shown in **Figure 4C**. Edge width is relative to correlation coefficients. We noted that ERBB3 and EPHA2 were most strongly associated with DDR1. Therefore, the correlation coefficients of mRNA expression and protein abundance between two genes and DDR1 in tumors were analyzed using heat maps (**Figure 4D**). ERBB2 is associated with angiogenesis, tumors metastasis, and drug resistance (38). There is also evidence that EPHA2 is linked to increased metastatic potential, poor prognosis, and lower survival rate (39). Considering the role of DDR1, we speculate that DDR1 may be involved in their regulatory mechanism.

### DDR1 expression correlates with immune cell infiltration in gastric cancer

**Figure 5:**
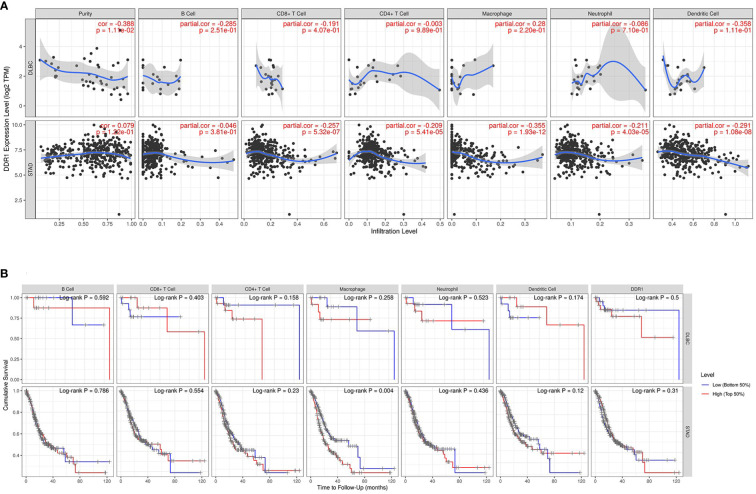
DDR1 expression is correlated with immune infiltration in STAD. **(A)** Correlation of DDR1 expression with immune infiltration levels in DLBC and STAD *via* TIMER database. DLBC serves as a control group. **(B)** Kaplan–Meier plots of the relationship between patients’ prognosis and immune cells infiltration in DLBC and STAD *via* TIMER database [blue line, low (bottom 50%); red line, high (top 50%)].

Immune cell infiltration has an irreplaceable role in independently predicting prognosis and lymph node metastasis status (40). Therefore, TIMER database was further applied to investigate the effect of DDR1 on the infiltration status of various TIICs in 39 cancer types and subtypes (**Supplementary Table S2**; **Supplementary Figures S4**–**6**). Subsequently, we counted that DDR1 was significantly associated with the tumor purity in 22 types and subtypes of cancer in total and correlated with B-cell infiltration in 12 types and subtypes of cancer, CD8^+^ T-cell infiltration in 13 types and subtypes of cancer, CD4^+^ T-cell infiltration in 17 types and subtypes of cancer, macrophage infiltration in 18 types and subtypes of cancer, neutrophil infiltration in 17 types and subtypes of cancer, and dendritic cell (DC) infiltration in 15 types and subtypes of cancer, respectively (p < 0.05) (**Supplementary Table S2**). Concretely, high DDR1 level was significantly and negatively linked to the infiltration of all the above TIICs in STAD, especially CD8^+^ T cells (r = −0.257, p = 5.32e−07), macrophages (r = −0.355, p = 1.93e−12), and DCs (r = −0.291, p = 1.08e−08) (**Figure 5A**). However, in DLBC, no significant association between DDR1 and any TIICs was observed. Moreover, we drew Kaplan–Meier plots for different TIICs to visualize the survival differences in STAD using TIMER database, with DLBC serving as a control group. It was only observed that macrophage infiltration was significantly associated to STAD prognosis (p = 0.004), while no significant association was noted in DLBC (**Figure 5B**). Generally, DDR1 level is significantly and negatively associated with immune infiltration in STAD, revealing that DDR1 plays a specific role in gastric cancer through immune cell infiltration, especially CD8^+^ T cells, macrophages, and DCs.

### Correlation between DDR1 expression and various immune markers

To better understand the role of DDR1 in the immune response, the relationships between DDR1 and various markers of diverse TIICs in STAD were further investigated *via* TIMER database (**Table 2**). Among 39 types of cancer analyzed by TIMER database, we found that DDR1 had no significant correlation (p<0.005) with six types of TIIC in DLBC, SKCM, and PAAD, which could be better used as the control. In the subsequent correlation analysis of immune markers, DLBC also showed a more significant difference from STAD. Here, we chose DLBC as the control group in order to better highlight the relationship between DDR1 and immune infiltration in STAD. With the adjustment based on purity, the correlation analysis in STAD revealed that DDR1 was closely linked to most of immune markers in various TIICs, such as CD3E of general T cells, CD86 of monocytes, CCL2 of tumor-associated macrophages (TAMs), and CCR7 of neutrophils. However, there were just four immune markers correlated to the DDR1 expression in DLBC (p < 0.01) (**Table 2**).

**Table 2:** Correlation between DDR1 level and markers of immune cells in STAD and DLBC *via* TIMER database.

Description	Gene markers	STAD				DLBC			
		None		Purity		None		Purity	
		Cor	P	Cor	P	Cor	P	Cor	P
**CD8^+^ T cell**	CD8A	−0.209	***	−0.220	***	−0.544	**	0.196	0.213
	CD8B	−0.225	***	−0.121	0.018	−0.411	*	0.183	0.246
**T cell (general)**	CD3D	−0.302	***	−0.315	***	−0.709	***	0.135	0.394
	CD3E	−0.241	***	−0.335	***	−0.750	***	0.145	0.358
	CD2	−0.257	***	−0.303	***	−0.737	***	0.135	0.394
**B cell**	CD19	−0.232	***	−0.218	***	0.145	0.361	−0.045	0.778
	CD79A	−0.252	***	−0.268	***	0.032	0.841	−0.080	0.612
**Monocyte**	CD86	−0.202	***	−0.286	***	−0.385	0.012	0.177	0.261
	CD115 (CSF1R)	−0.154	*	−0.208	***	−0.514	**	0.173	0.272
**TAM**	CCL2	−0.253	***	−0.205	***	−0.252	0.107	0.326	0.036
	CD68	0.072	0.146	−0.159	*	−0.410	*	0.035	0.827
	IL10	−0.165	**	−0.254	***	−0.211	0.180	0.113	0.476
**M1 Macrophage**	INOS (ISYNA1)	0.156	*	−0.009	0.863	−0.056	0.725	−0.090	0.570
	IRF5	0.178	**	−0.111	0.030	−0.257	0.100	0.020	0.901
	COX2 (PTGS2)	0.010	0.837	−0.126	0.014	−0.324	0.036	0.629	***
**M2 Macrophage**	CD163	−0.059	0.234	−0.190	**	−0.084	0.597	0.154	0.328
	VSIG4	−0.141	*	−0.166	*	−0.157	0.319	0.114	0.470
	MS4A4A	−0.242	***	−0.191	**	−0.202	0.200	0.189	0.230
**Neutrophils**	CD66b (CEACAM8)	−0.052	0.293	0.021	0.689	−0.273	0.080	0.199	0.207
	CD11b (ITGAM)	−0.035	0.475	−0.164	*	−0.309	0.046	0.210	0.182
	CCR7	−0.251	***	−0.292	***	−0.498	**	0.367	0.017
**NK cell**	KIR2DL1	−0.183	**	−0.077	0.137	−0.352	0.022	0.305	0.050
	KIR2DL3	−0.140	*	−0.132	0.010	−0.424	*	0.411	*
	KIR2DL4	−0.024	0.622	−0.165	*	−0.206	0.191	0.242	0.122
	KIR3DL1	−0.156	*	−0.124	0.016	−0.285	0.067	0.108	0.496
	KIR3DL2	−0.175	**	−0.161	*	−0.612	***	0.348	0.024
	KIR3DL3	0.044	0.369	−0.020	0.703	−0.117	0.461	0.122	0.440
	KIR2DS4	−0.131	*	−0.122	0.018	−0.239	0.127	0.092	0.560
DC	HLA-DPB1	−0.206	***	−0.293	***	−0.207	0.188	−0.185	0.240
	HLA-DQB1	−0.078	0.113	−0.282	***	−0.160	0.311	0.111	0.482
	HLA-DRA	−0.132	*	−0.276	***	−0.195	0.215	0.079	0.619
	HLA-DPA1	−0.135	*	−0.276	***	−0.303	0.051	−0.064	0.686
	BDCA-1 (CD1C)	−−0.274	***	−0.285	***	−0.026	0.872	−0.199	0.205
	BDCA-4 (NRP1)	−−0.142	*	−0.173	**	−0.263	0.092	0.337	0.030
	CD11c (ITGAX)	−0.063	0.204	−0.224	***	−0.533	**	0.127	0.422
Th1	T-bet (TBX21)	−0.190	***	−0.254	***	−−0.706	***	0.371	0.016
	STAT4	−0.263	***	−0.245	***	−0.732	***	0.144	0.360
	STAT1	0.156	*	−0.104	0.042	−0.451	*	0.206	0.190
	IFN-γ (IFNG)	−0.054	0.274	−0.190	**	−0.537	**	0.166	0.292
	TNF-α (TNF)	0.101	0.041	−0.281	***	−0.326	0.035	0.150	0.343
Th2	GATA3	−0.201	***	−0.174	**	−0.688	***	0.134	0.395
	BCL6	−0.026	0.599	−0.071	0.049	−0.277	0.011	0.301	*
	IL21	−0.105	0.032	0.011	0.836	0.065	0.684	0.181	0.250
	STAT6	0.201	***	−0.132	0.010	−0.418	*	0.259	0.098
	STAT5A	0.029	0.557	−0.002	0.971	−0.298	0.055	0.377	0.014
	IL13	−0.042	0.393	−0.135	*	0.181	0.251	−0.097	0.541
Th17	STAT3	0.152	***	−0.136	*	−0.410	*	0.198	0.209
	IL17A	−0.007	0.894	−0.122	0.017	−0.508	**	0.293	0.060
Treg	FOXP3	−0.024	0.630	−0.241	***	−0.633	***	0.337	0.030
	CCR8	−0.028	0.563	−0.168	*	−0.477	*	0.285	0.068
	STAT5B	−0.034	0.489	−0.023	0.661	−0.323	0.037	0.307	0.049
	TGFβ (TGFB1)	−0.061	0.217	−0.169	**	−0.517	**	0.409	*
**T cell exhaustion**	PD-1 (PDCD1)	−0.084	0.087	−0.175	**	−0.533	**	−0.060	0.704
	CTLA4	−0.087	0.077	−0.197	**	−0.702	***	0.133	0.401
	LAG3	−0.076	0.122	−0.227	***	−0.560	**	0.160	0.312
	TIM-3 (HAVCR2)	−0.127	*	−0.245	***	−0.387	0.011	0.097	0.542
	GZMB	−0.053	0.280	−0.254	***	−0.242	0.122	0.075	0.637

*p < 0.01; **p < 0.001; ***p < 0.0001. TAM, tumor-associated macrophage.

**Figure 6:**
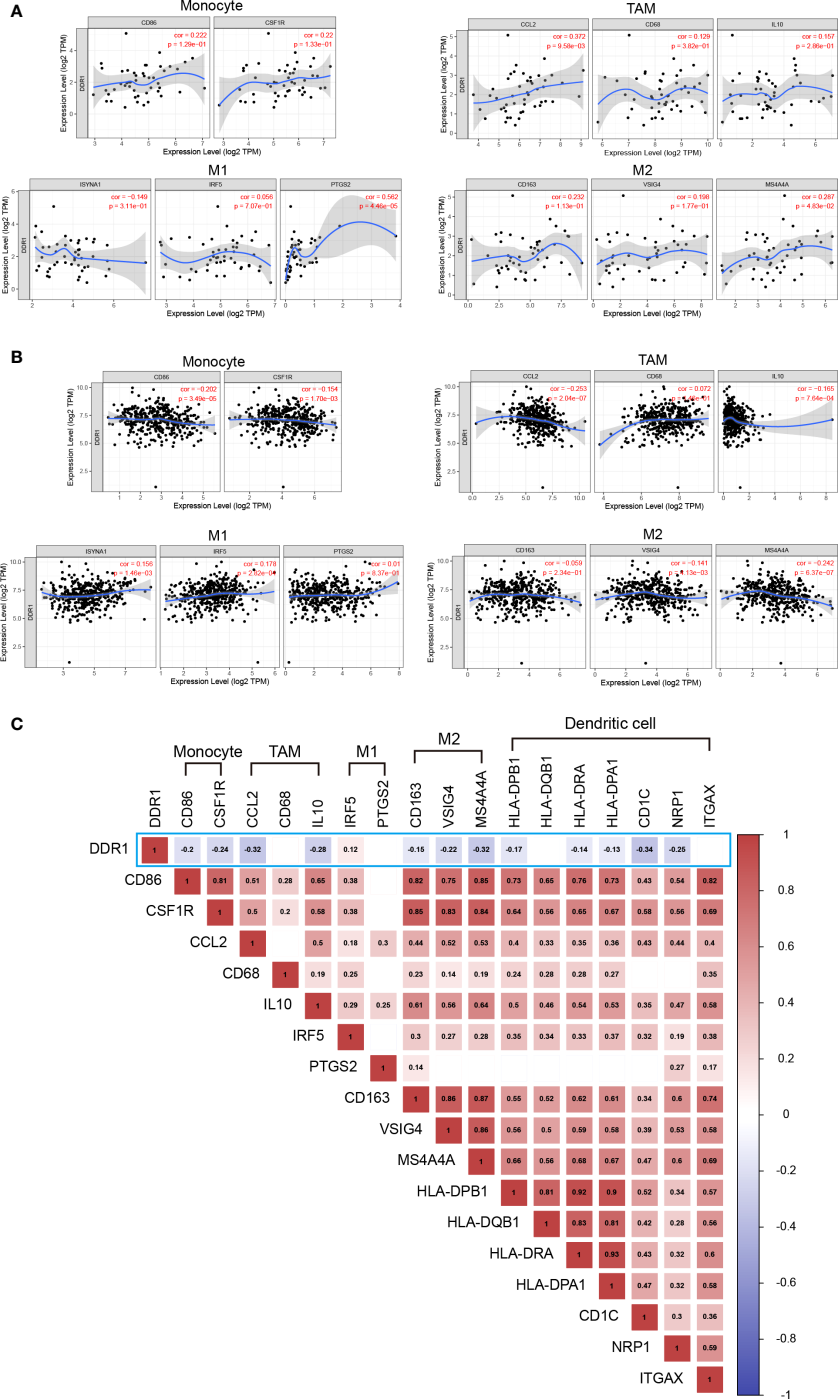
Correlation between DDR1 expression level and immune markers in STAD and DLBC. **(A)** Scatterplots of correlation between DDR1 expression level and immunological marker sets of monocytes, TAMs, and M1 and M2 macrophages in DLBC *via* TIMER database. **(B)** Scatterplots of correlation between DDR1 expression level and immunological marker sets of monocytes, TAMs, and M1 and M2 macrophages in STAD *via* TIMER database. **(C)** Correlation heatmap of DDR1 expression level and immunological marker sets of monocytes, TAMs, M1 macrophages, M2 macrophages, and dendritic cells based on 407 STAD samples from TCGA. (Blank indicates the correlation is not significant. The p-value cutoff is 0.05.).

Interestingly, we noted the significant correlation between DDR1 level and Treg and T cell exhaustion markers like CCR8, PD-1, CTLA-4, and TIM-3 (**Table 2**), revealing that DDR1 might play a potential role in immune escape in STAD, but further studies are needed about its mechanisms. Furthermore, the DDR1 level was significantly correlated to the majority of monocyte, TAM, and M2 macrophage immune markers in STAD, such as CD86, CCL2, and MS4A4A. To show the relationship between them visually, we thus generated the expression scatterplots in STAD using TIMER database, with DLBC serving as a control group in like manner (**Figures 6A, B**). Subsequently, the GEPIA database was used to confirm the relationships between DDR1 level and the above monocyte, and TAM, M1, and M2 macrophage markers (**Table 3**). Just as expected, the correlations in GEPIA corroborated with the previous results. In addition, we directly used 407 STAD samples from TCGA database to calculate the Spearman correlation coefficient of DDR1 and various immune markers (**Figure 6C**). It also turned out that DDR1 had a significant negative correlation with most immune markers of monocytes, TAMs, M2 macrophages, and DCs, while there was no significant correlation with M1 macrophage markers like PTGS2, or a significantly positive association like IRF5. Therefore, DDR1 may be correlated to regulating the polarization of macrophages in STAD. Simultaneously, its significant correlations with DC markers revealed the significant correlation between DDR1 and DC infiltration. Therefore, these findings validate that DDR1 is involved in immune infiltration and immune escape in gastric cancer.

**Table 3:** Correlation between DDR1 level and immune markers of monocyte, TAM, and macrophages of STAD *via* GEPIA database.

Description	Gene markers	STAD			
		Tumor		Normal	
		R	p	R	p
**Monocyte**	CD86	−0.19	**	0.22	0.20
	CD115 (CSF1R)	−0.11	0.029	0.13	0.46
**TAM**	CCL2	−0.21	***	−0.52	*
	CD68	0.094	0.058	0.53	*
	IL10	−0.13	*	0.038	0.82
**M1 Macrophage**	INOS (ISYNA1)	0.16	*	−0.21	0.21
	IRF5	0.24	***	0.69	***
	COX2 (PTGS2)	0.034	0.49	−0.45	*
**M2 Macrophage**	CD163	−0.12	0.016	−0.42	0.012
	VSIG4	−0.11	0.027	−0.31	0.069
	MS4A4A	−0.20	***	−0.54	**

*p < 0.01; **p < 0.001; ***p < 0.0001.

## Discussion

Gastric cancer is one of the common malignant tumors worldwide. Due to the difficulty of its early diagnosis and the high late recurrence rate, gastric cancer has poor prognosis and high mortality (41). With the deepening of research, it has been found that gastric cancer, especially in advanced stage, has a strong ability of metastasis and invasion. Inhibition of metastasis thus becomes an essential step in treating gastric cancer. In recent years, more and more researchers have turned their attention to cancer immunotherapy. At present, immunotherapy, such as ICIs, has become a first-line treatment for many advanced cancers. In spite of the fact that ICIs have good efficacy in treating malignant tumors, their application is limited in gastric cancer. A limitation of the application of antibodies is the low or non-response rate in some patients. Promoting intratumoral T-cell infiltration is known to significantly increase the efficacy of PD-1 antibody (42, 43). Therefore, investigating the mechanisms underlying immune cell infiltration is essential to improve the efficacy of ICIs for gastric cancer.

On the basis of previous screening studies, our study selected DDR1 as a target to investigate its role in gastric cancer. As a receptor for collagen tyrosine kinase, DDR1 is a major component of the ECM (44). Previous studies demonstrated that DDR1 was overexpressed and linked to invasion and metastasis in a variety of cancers, such as gastric, bladder, and other cancers (18, 45, 46). A recent study also showed that ECD of DDR1 was associated with immune infiltration of tumors (21). Consequently, we studied data from multiple databases to understand the effect of DDR1 in gastric cancer. Our analysis revealed that the expression of DDR1 was upregulated in gastric cancer (**Figure 1**). Prognostic analysis conducted by PrognoScan, Kaplan–Meier plotter, and GEPIA databases also suggested that DDR1 affected the prognosis of patients with various types of cancer to varying degrees (**Figure 3**). These data from multiple sources reflected that the high expression of DDR1 led to a significantly poorer prognosis in gastric cancer patients. Moreover, studies based on gender, tumor–node–metastasis (TNM) stages, Lauren classification, and other clinicopathological characteristics also proved the clinical prognostic value of DDR1 in the treatment of gastric cancer. The results showed that DDR1 was significantly correlated with a variety of clinicopathological characteristics in STAD (**Table 1**). Notably, high expression of DDR1 in N2 and M1 stages of gastric cancer had comparatively high HR values in the prognostic analysis, revealing the crucial role of DDR1 in local lymph node metastasis and distant metastasis of gastric cancer. Therefore, we suggest that DDR1 has good prognostic value as a potential tumor therapeutic target in patients with gastric cancer, thereby effectively promoting the development of precision therapy for gastric cancer. Specially, targeting DDR1 is suggested to have a good therapeutic potential for metastatic advanced gastric cancer.

Subsequently, this study further investigated the underlying molecular mechanism of DDR1. We found that DDR1 was closely related to SHC1, PTPN11, TM4SF1, and so on by using STRING. In particular, we found that DDR1 interacted with PPP1R1B. PPP1R1B can modulate downstream signaling of various kinases in pancreatic cancer by regulating proteins phosphatase 1 activity, which in turn regulates the activity of many phosphorylated proteins (47). Among them, the regulation of PPP1R1B induces phosphorylation of Mdm2 Ser166 and promotes the degradation of p53. In addition, DDR1 can regulate p53 *via* the positive feedback of DDR1-RAS/MAPK-p53-P21 module (48). Therefore, we proposed a hypothesis that DDR1 interacts with PPP1R1B through the regulation of p53. It would be worthwhile to explore this further. Then, DDR1-interacting genes and proteins were also investigated using the PINA database. The majority of these genes are associated with metastasis and invasion of cancer. The obvious correlation between DDR1 and EBRR2 and the mechanism of action also deserves our further study. In addition, after referring to the analysis of DDR1-related genes in many references and databases, we performed the pathway enrichment analysis of DDR1 and its related genes (9, 16). Pathways were mainly enriched in MET signaling and PID ERBB2 ERBB3 pathway. Here, we also consider that the number of genes that we screened is not large, so the analysis of related pathways also needs further validation.

Previous studies have revealed some key mechanisms of DDR1 in immune infiltration (21, 22). In the present study, we investigated the infiltration situation based on DDR1 in gastric cancer. The results showed that DDR1 expression significantly affected the infiltration of various TIICs in gastric cancer, especially CD8^+^ cells, macrophages, and DCs (**Figure 5A**). Thus, DDR1 is reasonably supposed to be involved in macrophage polarization and T-cell activation regulated by DCs and therefore affects immune infiltration. In addition, the prognostic analysis of different TIICs showed that macrophage infiltration significantly correlated with the survival of gastric cancer patients (**Figure 5B**). Following the assessment of overall infiltration in gastric cancer, we further explored the correlation between DDR1 and various immune cell markers (**Figure 6**; **Table 2**). The significant correlation between DDR1 and regulatory T cells and T-cell exhaustion markers such as TGFβ1, PD-1, and CTLA-4 indicated that DDR1 was involved in immune escape and tumor invasion. Moreover, obvious associations between immune markers of T-helper cells and DDR1 were observed as well, such as tumor necrosis factor alpha (TNF-α) of Th1, GATA3 of Th2, and STAT3 of Th17. These results imply the crucial role that DDR1 played in the recruitment, effect, and regulation of various TIICs in gastric cancer.

In summary, this study deepens our understanding of the various roles of DDR1 in the progression of gastric cancer and also demonstrates the potential clinical value of DDR1 as a therapeutic target for gastric cancer. However, there are still limitations in our study. In general, our study principally focused on mRNA levels, and there was not enough data based on protein level to analyze. In addition, we mainly focalize the infiltration study on STAD but lacked the study on other rare subtypes of gastric cancer, such as squamous cell carcinoma of the stomach. In addition, even though there is a significant and negative correlation between DDR1 and many immune markers of various TIICs in gastric cancer, the correlation is not very strong, which also reflects the complexity of the mechanism of immune infiltration. Therefore, the molecular mechanisms and signaling pathways of DDR1 affecting immune infiltration and escape, tumor invasion, and metastasis also remain to be further studied. Overall, our study demonstrates the multiple potentials of DDR1 in the immunotherapy of gastric cancer, including immune infiltration and tumor invasion, and also expands the direction of DDR1 signaling mechanism research. Moreover, all new discoveries of DDR1 may provide a new strategy for improving the efficacy of ICI therapy.

## Data availability statement

The datasets presented in this study can be found in online repositories. The names of the repository/repositories and accession number(s) can be found in the article/**Supplementary Material**.

## Author contributions

LY, SW, and YF contributed to the conception of the study. DP, HC, and KK contributed the data collection. SW, YF, and KK performed the data analysis. SW and YF wrote the manuscript. YZ, XH, and LY helped perform the analysis with constructive discussions. All authors contributed to article and approved the submitted version.

## Funding

This work was supported by Scientific and Innovative Action Plan of Shanghai (no. 20S11901600 and 18431902800) and National Natural Science Foundation of China (no. 81572979).

## Conflict of interest

The authors declare that the research was conducted in the absence of any commercial or financial relationships that could be construed as a potential conflict of interest.

## Publisher’s note

All claims expressed in this article are solely those of the authors and do not necessarily represent those of their affiliated organizations, or those of the publisher, the editors and the reviewers. Any product that may be evaluated in this article, or claim that may be made by its manufacturer, is not guaranteed or endorsed by the publisher.
