# Supplementary figures and images for: Discoidin domain receptor 1 is a potential target correlated with tumor invasion and immune infiltration in gastric cancer
Source: Front Immunol. 2022 Jul 22;13:933165. doi: 10.3389/fimmu.2022.933165 (PMC9353406; doi:10.3389/fimmu.2022.933165)

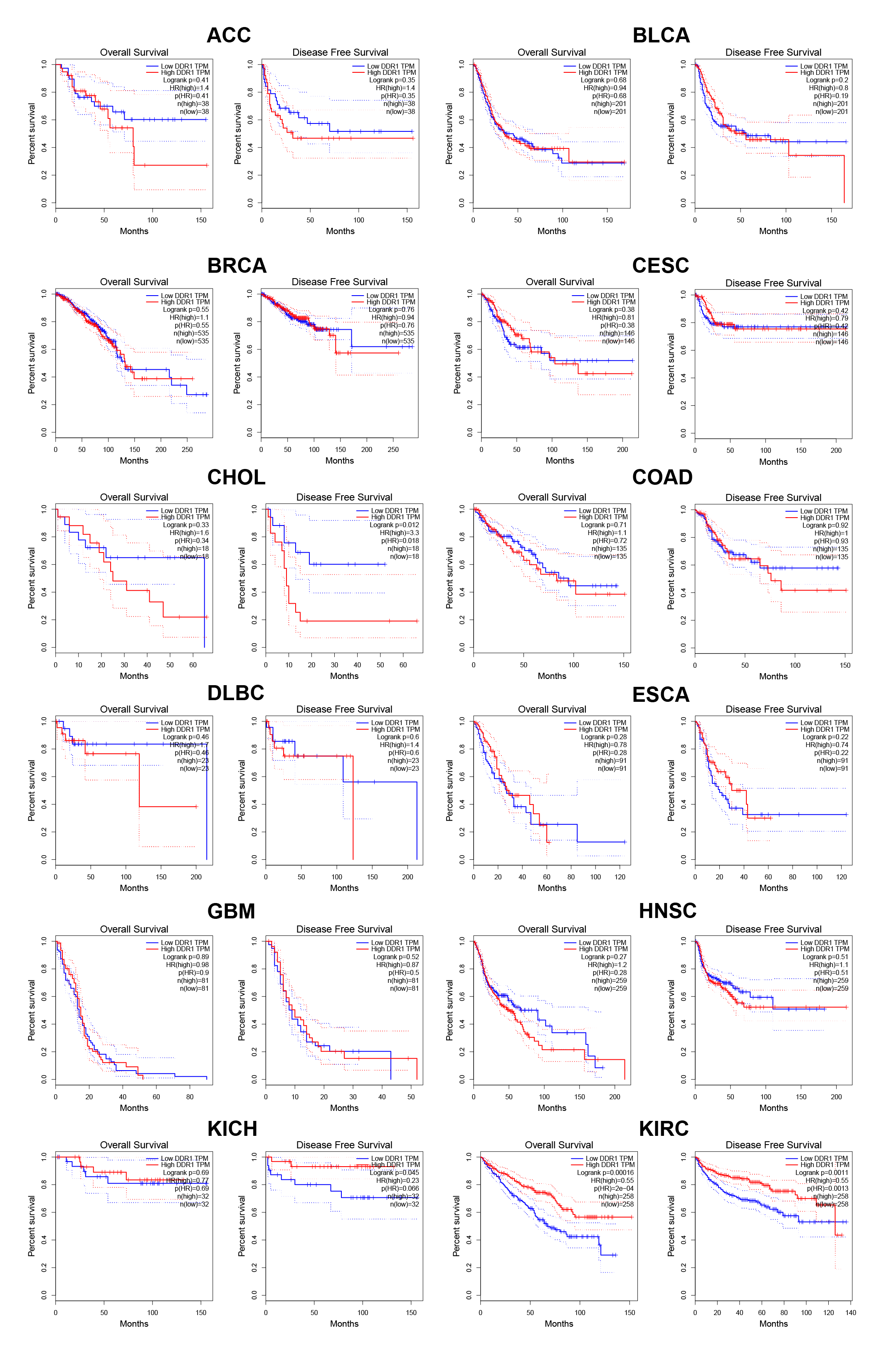

Supplement: Supplementary file 1 [file Image_1.tif]

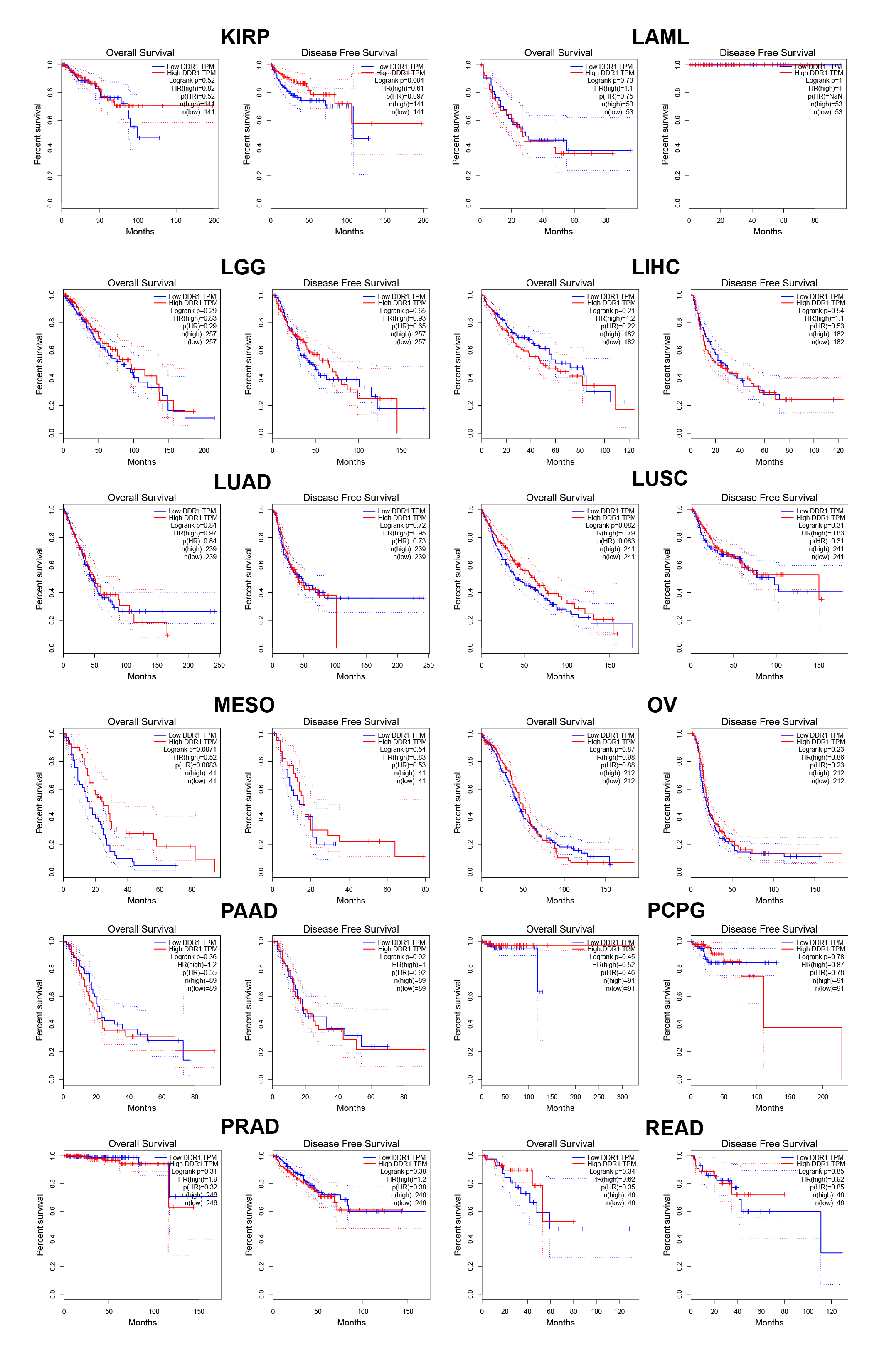

Supplement: Supplementary file 2 [file Image_2.tif]

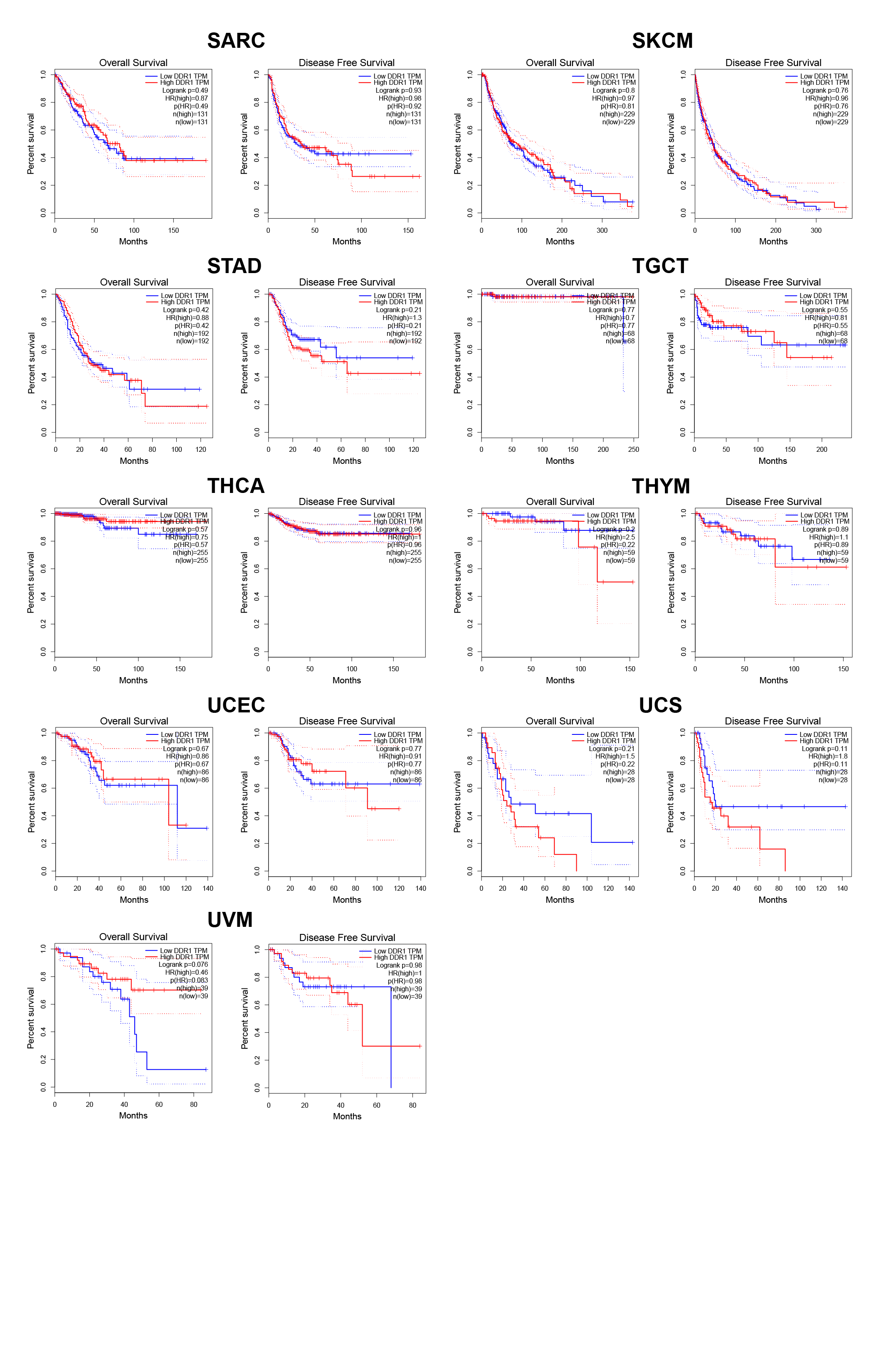

Supplement: Supplementary file 3 [file Image_3.tif]
