# Supplementary material for: Discoidin domain receptor 1 is a potential target correlated with tumor invasion and immune infiltration in gastric cancer
Source: Front Immunol. 2022 Jul 22;13:933165. doi: 10.3389/fimmu.2022.933165 (PMC9353406; doi:10.3389/fimmu.2022.933165)
Supplement: Supplementary file 7 [file DataSheet_1.docx]

Supplementary Material

# Supplementary Figures and Tables

## Supplementary Figures

**Supplementary Figure 1.** Kaplan-Meier survival curves according to the expression of DDR1 in ACC, BLCA, BRCA, CESC, CHOL, COAD, DLBC, ESCA, GBM, HNSC, KICH, KIRC in the GEPIA database. The end points are OS and DFS for each type of cancer.

**Supplementary Figure 2** Kaplan-Meier survival curves according to the expression of DDR1 in KIRP, LAML, LGG, LIHC, LUAD, LUSC, MESO, OV, PAAD, PCPG, PRAD, READ in the GEPIA database. The end points are OS and DFS for each type of cancer.

**Supplementary Figure 3** Kaplan-Meier survival curves according to the expression of DDR1 in SARC, SKCM, STAD, TGCT, THCA, THYM, UCEC, UCS, UVM in the GEPIA database. The end points are OS and DFS for each type of cancer.

**Supplementary Figure 4** Scatterplots for the correlation of DDR1 expression with immune infiltration level in ACC, BLCA, BRCA, BRCA-Basal, BRCA-Her2, BRCA-Luminal, CESC, CHOL, COAD, DLBC, ESCA, GBM, HNSC via TIMER database.

**Supplementary Figure 5** Scatterplots for the correlation of DDR1 expression with immune infiltration level in HNSC-HPVpos, HNSC-HPVneg, KICH, KIRC, KIRP, LGG, LIHC, LUAD, LUSC, MESO, OV, PAAD, PCPG via TIMER database.

**Supplementary Figure 6** Scatterplots for the correlation of DDR1 expression with immune infiltration level in PRAD, READ, SARC, SKCM, SKCM-Primary, SKCM-Metastasis, STAD, TGCT, THCA, THYM, UCEC, UCS, UVM via TIMER database.

## Supplementary Tables

**Supplementary Table 1.** DDR1 expression levels in different cell lines of 29 cancer types using GEDS platform.

|  | Cancer types | symbol | Cell line numbers | expression |
| --- | --- | --- | --- | --- |
| 1 | adrenal cortex | DDR1 | 1 | 6.23631 |
| 2 | autonomic ganglia | DDR1 | 23 | 7.066118261 |
| 3 | biliary tract | DDR1 | 11 | 30.48836818 |
| 4 | bone | DDR1 | 42 | 8.644647143 |
| 5 | brain | DDR1 | 1 | 3.58231 |
| 6 | breast | DDR1 | 58 | 46.34835483 |
| 7 | central nervous system | DDR1 | 73 | 19.09470562 |
| 8 | cervix | DDR1 | 8 | 17.97260875 |
| 9 | endometrium | DDR1 | 28 | 25.99185429 |
| 10 | eye | DDR1 | 1 | 7.24986 |
| 11 | haematopoietic and lymphoid | DDR1 | 190 | 1.773921158 |
| 12 | intestine | DDR1 | 61 | 28.94551787 |
| 13 | kidney | DDR1 | 37 | 13.00709892 |
| 14 | liver | DDR1 | 25 | 13.9190416 |
| 15 | lung | DDR1 | 202 | 22.48656005 |
| 16 | oesophagus | DDR1 | 29 | 45.26897862 |
| 17 | ovary | DDR1 | 55 | 22.03701673 |
| 18 | pancreas | DDR1 | 49 | 32.13460714 |
| 19 | placenta | DDR1 | 1 | 17.0496 |
| 20 | pleura | DDR1 | 9 | 8.825978889 |
| 21 | prostate | DDR1 | 8 | 24.75663625 |
| 22 | salivary gland | DDR1 | 2 | 52.104005 |
| 23 | skin | DDR1 | 61 | 14.95974262 |
| 24 | soft tissue | DDR1 | 18 | 10.91538056 |
| 25 | stomach | DDR1 | 37 | 28.96382676 |
| 26 | synovium | DDR1 | 1 | 10.72717 |
| 27 | thyroid | DDR1 | 12 | 13.10082417 |
| 28 | upper aerodigestive tract | DDR1 | 34 | 59.00998971 |
| 29 | urinary tract | DDR1 | 37 | 33.75796 |

**Supplementary Table 2.** Correlation of DDR1 expression with immune infiltration level in 39 different types of cancer via TIMER database, including purity-corrected partial Spearman’s rho value and statistical significance.

| cancer | variable | partial.cor | p |
| --- | --- | --- | --- |
| ACC | Purity | 0.285661665 | 0.013618835 |
|  | B Cell | 0.397983042 | 0.000488645 |
|  | CD8+ T Cell | 0.303837523 | 0.008967858 |
|  | CD4+ T Cell | 0.216611631 | 0.065664428 |
|  | Macrophage | 0.366397323 | 0.001432024 |
|  | Neutrophil | 0.304809673 | 0.00874002 |
|  | Dendritic Cell | 0.285984976 | 0.014177057 |
| BLCA | Purity | 0.162743814 | 0.001709567 |
|  | B Cell | 0.154973509 | 0.003072766 |
|  | CD8+ T Cell | 0.058186155 | 0.26686985 |
|  | CD4+ T Cell | -0.059271455 | 0.258691749 |
|  | Macrophage | -0.043738938 | 0.404752375 |
|  | Neutrophil | 0.082039662 | 0.118687661 |
|  | Dendritic Cell | -0.056999221 | 0.277427425 |
| BRCA | Purity | 0.170376797 | 6.40E-08 |
|  | B Cell | -0.038117249 | 0.23391351 |
|  | CD8+ T Cell | -0.065778431 | 0.039919988 |
|  | CD4+ T Cell | 0.021620633 | 0.502988693 |
|  | Macrophage | -0.107104523 | 0.000769785 |
|  | Neutrophil | -0.032805694 | 0.312201745 |
|  | Dendritic Cell | -0.055738125 | 0.085806957 |
| BRCA-Basal | Purity | 0.244406645 | 0.00524854 |
|  | B Cell | -0.016797564 | 0.853096929 |
|  | CD8+ T Cell | 0.100690786 | 0.267804967 |
|  | CD4+ T Cell | 0.108986285 | 0.232101255 |
|  | Macrophage | -0.01415542 | 0.87449295 |
|  | Neutrophil | 0.222749879 | 0.019335411 |
|  | Dendritic Cell | 0.214706438 | 0.02179077 |
| BRCA-Her2 | Purity | 0.236511693 | 0.071310848 |
|  | B Cell | -0.002306733 | 0.986288915 |
|  | CD8+ T Cell | 0.02358841 | 0.861733998 |
|  | CD4+ T Cell | 0.21114159 | 0.111610375 |
|  | Macrophage | 0.079477651 | 0.553149805 |
|  | Neutrophil | 0.10934343 | 0.41389347 |
|  | Dendritic Cell | -0.014484465 | 0.915619952 |
| BRCA-Luminal | Purity | 0.149898336 | 0.000440633 |
|  | B Cell | -0.035246006 | 0.413266274 |
|  | CD8+ T Cell | -0.061333714 | 0.155803808 |
|  | CD4+ T Cell | 0.034792551 | 0.421472835 |
|  | Macrophage | -0.171476638 | 6.00E-05 |
|  | Neutrophil | -0.057987904 | 0.180897757 |
|  | Dendritic Cell | -0.066808331 | 0.122735811 |
| CESC | Purity | 0.0307641 | 0.609526261 |
|  | B Cell | 0.035121836 | 0.560512695 |
|  | CD8+ T Cell | -0.06602758 | 0.276090711 |
|  | CD4+ T Cell | 0.212839324 | 0.000360688 |
|  | Macrophage | -0.142438799 | 0.017690127 |
|  | Neutrophil | 0.183784042 | 0.002132392 |
|  | Dendritic Cell | 0.059479346 | 0.324853813 |
| CHOL | Purity | 0.484037091 | 0.002779365 |
|  | B Cell | 0.00368065 | 0.983258222 |
|  | CD8+ T Cell | 0.068669164 | 0.695086653 |
|  | CD4+ T Cell | 0.205062344 | 0.237318655 |
|  | Macrophage | 0.235116601 | 0.173956275 |
|  | Neutrophil | 0.208722317 | 0.228867847 |
|  | Dendritic Cell | 0.055870552 | 0.74989363 |
| COAD | Purity | 0.042886898 | 0.388166793 |
|  | B Cell | -0.01122153 | 0.822089606 |
|  | CD8+ T Cell | -0.086454444 | 0.081877144 |
|  | CD4+ T Cell | 0.307222124 | 3.10E-10 |
|  | Macrophage | 0.100149621 | 0.044240416 |
|  | Neutrophil | 0.081728946 | 0.102207042 |
|  | Dendritic Cell | 0.157352529 | 0.001551779 |
| DLBC | Purity | -0.38803553 | 0.011109651 |
|  | B Cell | -0.285286091 | 0.251166689 |
|  | CD8+ T Cell | -0.190971105 | 0.406977491 |
|  | CD4+ T Cell | -0.003144828 | 0.989205815 |
|  | Macrophage | 0.279628301 | 0.219587694 |
|  | Neutrophil | -0.086410464 | 0.709570424 |
|  | Dendritic Cell | -0.357599806 | 0.111489924 |
| ESCA | Purity | 0.285654 | 9.70E-05 |
|  | B Cell | -0.04934 | 0.511855 |
|  | CD8+ T Cell | -0.09657 | 0.197204 |
|  | CD4+ T Cell | -0.14323 | 0.055776 |
|  | Macrophage | -0.09217 | 0.218457 |
|  | Neutrophil | -0.17953 | 0.015889 |
|  | Dendritic Cell | -0.02182 | 0.771264 |
| GBM | Purity | 0.117953 | 0.015707 |
|  | B Cell | 0.110922 | 0.023328 |
|  | CD8+ T Cell | 0.153881 | 0.001602 |
|  | CD4+ T Cell | 0.273019 | 1.40E-08 |
|  | Macrophage | 0.132677 | 0.006598 |
|  | Neutrophil | 0.119034 | 0.014891 |
|  | Dendritic Cell | -0.03135 | 0.522662 |
| HNSC | Purity | 0.206677 | 3.70E-06 |
|  | B Cell | 0.0962 | 0.035889 |
|  | CD8+ T Cell | -0.05678 | 0.217253 |
|  | CD4+ T Cell | 0.260136 | 7.28E-09 |
|  | Macrophage | 0.053823 | 0.237733 |
|  | Neutrophil | 0.044529 | 0.330799 |
|  | Dendritic Cell | 0.06626 | 0.146356 |
| HNSC-HPVpos | Purity | -0.02281 | 0.831 |
|  | B Cell | 0.024221 | 0.832199 |
|  | CD8+ T Cell | 0.040995 | 0.721567 |
|  | CD4+ T Cell | 0.287069 | 0.008925 |
|  | Macrophage | -0.09186 | 0.400236 |
|  | Neutrophil | 0.035097 | 0.749811 |
|  | Dendritic Cell | 0.056414 | 0.612459 |
| HNSC-HPVneg | Purity | 0.236627 | 1.65E-06 |
|  | B Cell | 0.107309 | 0.033221 |
|  | CD8+ T Cell | -0.09228 | 0.067636 |
|  | CD4+ T Cell | 0.263114 | 1.12E-07 |
|  | Macrophage | 0.060113 | 0.233855 |
|  | Neutrophil | 0.03764 | 0.457987 |
|  | Dendritic Cell | 0.060174 | 0.232183 |
| KICH | Purity | 0.121853 | 0.329727 |
|  | B Cell | 0.225705 | 0.070639 |
|  | CD8+ T Cell | 0.223395 | 0.073646 |
|  | CD4+ T Cell | -0.25615 | 0.039435 |
|  | Macrophage | 0.208957 | 0.094826 |
|  | Neutrophil | -0.10361 | 0.411438 |
|  | Dendritic Cell | 0.138203 | 0.272253 |
| KIRC | Purity | 0.096367 | 0.038402 |
|  | B Cell | 0.163932 | 0.000421 |
|  | CD8+ T Cell | 0.029878 | 0.532856 |
|  | CD4+ T Cell | 0.143641 | 0.002012 |
|  | Macrophage | 0.174784 | 0.000201 |
|  | Neutrophil | 0.178281 | 0.000125 |
|  | Dendritic Cell | 0.113301 | 0.015609 |
| KIRP | Purity | 0.014974 | 0.810462 |
|  | B Cell | -0.11412 | 0.068303 |
|  | CD8+ T Cell | -0.18858 | 0.002353 |
|  | CD4+ T Cell | 0.073269 | 0.240903 |
|  | Macrophage | 0.023193 | 0.715721 |
|  | Neutrophil | 0.173844 | 0.005108 |
|  | Dendritic Cell | 0.110388 | 0.077908 |
| LGG | Purity | 0.209662 | 3.69E-06 |
|  | B Cell | 0.144587 | 0.001527 |
|  | CD8+ T Cell | 0.042211 | 0.357119 |
|  | CD4+ T Cell | 0.157502 | 0.000563 |
|  | Macrophage | 0.239911 | 1.28E-07 |
|  | Neutrophil | 0.054781 | 0.23339 |
|  | Dendritic Cell | 0.144788 | 0.001539 |
| LIHC | Purity | 0.018773 | 0.727873 |
|  | B Cell | 0.396038 | 2.29E-14 |
|  | CD8+ T Cell | 0.268563 | 4.62E-07 |
|  | CD4+ T Cell | 0.50347 | 1.66E-23 |
|  | Macrophage | 0.494178 | 2.13E-22 |
|  | Neutrophil | 0.401504 | 8.53E-15 |
|  | Dendritic Cell | 0.411476 | 2.52E-15 |
| LUAD | Purity | 0.151588 | 0.000724 |
|  | B Cell | 0.039102 | 0.390705 |
|  | CD8+ T Cell | -0.0894 | 0.048628 |
|  | CD4+ T Cell | 0.209093 | 3.49E-06 |
|  | Macrophage | 0.073591 | 0.105514 |
|  | Neutrophil | 0.017316 | 0.704237 |
|  | Dendritic Cell | 0.067021 | 0.139297 |
| LUSC | Purity | 0.198581 | 1.22E-05 |
|  | B Cell | -0.1151 | 0.012341 |
|  | CD8+ T Cell | -0.27199 | 1.68E-09 |
|  | CD4+ T Cell | 0.04266 | 0.353549 |
|  | Macrophage | -0.13448 | 0.003253 |
|  | Neutrophil | -0.15319 | 0.000799 |
|  | Dendritic Cell | -0.24449 | 7.01E-08 |
| MESO | Purity | 0.102523 | 0.347563 |
|  | B Cell | 0.121489 | 0.270954 |
|  | CD8+ T Cell | -0.25209 | 0.020704 |
|  | CD4+ T Cell | -0.12088 | 0.273391 |
|  | Macrophage | 0.059294 | 0.59212 |
|  | Neutrophil | -0.01865 | 0.866297 |
|  | Dendritic Cell | -0.30627 | 0.004606 |
| OV | Purity | 0.09178 | 0.04357 |
|  | B Cell | 0.07507 | 0.100437 |
|  | CD8+ T Cell | -0.06679 | 0.14399 |
|  | CD4+ T Cell | 0.031261 | 0.49443 |
|  | Macrophage | -0.18904 | 3.07E-05 |
|  | Neutrophil | -0.03096 | 0.498547 |
|  | Dendritic Cell | -0.01621 | 0.723143 |
| PAAD | Purity | 0.12647 | 0.098292 |
|  | B Cell | 0.057133 | 0.457946 |
|  | CD8+ T Cell | 0.079573 | 0.300874 |
|  | CD4+ T Cell | -0.06572 | 0.395925 |
|  | Macrophage | -0.08314 | 0.279662 |
|  | Neutrophil | -0.00555 | 0.942558 |
|  | Dendritic Cell | -0.03791 | 0.622526 |
| PCPG | Purity | 0.144281 | 0.062054 |
|  | B Cell | 0.041761 | 0.592065 |
|  | CD8+ T Cell | 0.095642 | 0.218883 |
|  | CD4+ T Cell | 0.123736 | 0.111129 |
|  | Macrophage | 0.234385 | 0.00237 |
|  | Neutrophil | 0.308739 | 4.92E-05 |
|  | Dendritic Cell | 0.152713 | 0.048811 |
| PRAD | Purity | -0.11984 | 0.014339 |
|  | B Cell | 0.328775 | 7.68E-12 |
|  | CD8+ T Cell | 0.417208 | 5.97E-19 |
|  | CD4+ T Cell | 0.133039 | 0.006915 |
|  | Macrophage | 0.405489 | 6.78E-18 |
|  | Neutrophil | 0.401781 | 1.71E-17 |
|  | Dendritic Cell | 0.299124 | 5.03E-10 |
| READ | Purity | 0.055638 | 0.513808 |
|  | B Cell | 0.035879 | 0.674981 |
|  | CD8+ T Cell | -0.01184 | 0.889938 |
|  | CD4+ T Cell | 0.124104 | 0.145507 |
|  | Macrophage | 0.235885 | 0.005182 |
|  | Neutrophil | -0.12231 | 0.152979 |
|  | Dendritic Cell | 0.087454 | 0.305968 |
| SARC | Purity | 0.346224 | 2.63E-08 |
|  | B Cell | 0.09 | 0.165478 |
|  | CD8+ T Cell | -0.0414 | 0.523248 |
|  | CD4+ T Cell | -0.23734 | 0.000213 |
|  | Macrophage | -0.24554 | 0.000143 |
|  | Neutrophil | -0.03392 | 0.599489 |
|  | Dendritic Cell | -0.24116 | 0.000157 |
| SKCM | Purity | 0.181694 | 9.21E-05 |
|  | B Cell | -0.00872 | 0.85395 |
|  | CD8+ T Cell | -0.03107 | 0.51668 |
|  | CD4+ T Cell | 0.037068 | 0.434865 |
|  | Macrophage | -0.00963 | 0.837975 |
|  | Neutrophil | 0.015953 | 0.735181 |
|  | Dendritic Cell | -0.00696 | 0.883533 |
| SKCM-Primary | Purity | 0.143939 | 0.146904 |
|  | B Cell | -0.08336 | 0.407228 |
|  | CD8+ T Cell | 0.062243 | 0.536347 |
|  | CD4+ T Cell | 0.043218 | 0.667827 |
|  | Macrophage | 0.046403 | 0.644952 |
|  | Neutrophil | 0.124164 | 0.218397 |
|  | Dendritic Cell | -0.08542 | 0.395716 |
| SKCM-Metastasis | Purity | 0.187116 | 0.000393 |
|  | B Cell | 0.038661 | 0.473494 |
|  | CD8+ T Cell | -0.03514 | 0.520931 |
|  | CD4+ T Cell | 0.045484 | 0.400371 |
|  | Macrophage | -0.00195 | 0.970968 |
|  | Neutrophil | 0.019474 | 0.716166 |
|  | Dendritic Cell | 0.027263 | 0.614321 |
| STAD | Purity | 0.079366 | 0.122474 |
|  | B Cell | -0.04574 | 0.380987 |
|  | CD8+ T Cell | -0.25717 | 5.32E-07 |
|  | CD4+ T Cell | -0.20941 | 5.41E-05 |
|  | Macrophage | -0.35518 | 1.93E-12 |
|  | Neutrophil | -0.21146 | 4.03E-05 |
|  | Dendritic Cell | -0.29136 | 1.08E-08 |
| TGCT | Purity | 0.324042 | 5.88E-05 |
|  | B Cell | -0.05716 | 0.491625 |
|  | CD8+ T Cell | -0.02483 | 0.765281 |
|  | CD4+ T Cell | 0.28239 | 0.000554 |
|  | Macrophage | 0.198253 | 0.016079 |
|  | Neutrophil | 0.077004 | 0.353907 |
|  | Dendritic Cell | 0.161398 | 0.051631 |
| THCA | Purity | -0.02848 | 0.529781 |
|  | B Cell | 0.426099 | 1.11E-22 |
|  | CD8+ T Cell | -0.46693 | 9.68E-28 |
|  | CD4+ T Cell | 0.424053 | 1.02E-22 |
|  | Macrophage | 0.306507 | 4.49E-12 |
|  | Neutrophil | 0.195606 | 1.35E-05 |
|  | Dendritic Cell | 0.124783 | 0.005928 |
| THYM | Purity | 0.130997 | 0.161021 |
|  | B Cell | -0.26399 | 0.004538 |
|  | CD8+ T Cell | -0.24321 | 0.009121 |
|  | CD4+ T Cell | -0.58077 | 2.36E-11 |
|  | Macrophage | -0.07529 | 0.42597 |
|  | Neutrophil | 0.231074 | 0.013377 |
|  | Dendritic Cell | -0.48938 | 3.29E-08 |
| UCEC | Purity | 0.061119 | 0.296264 |
|  | B Cell | -0.02262 | 0.701726 |
|  | CD8+ T Cell | -0.09708 | 0.099553 |
|  | CD4+ T Cell | -0.05244 | 0.372792 |
|  | Macrophage | -0.08648 | 0.140432 |
|  | Neutrophil | 0.202292 | 0.000494 |
|  | Dendritic Cell | 0.160262 | 0.006059 |
| UCS | Purity | -0.12265 | 0.376948 |
|  | B Cell | -0.0078 | 0.955786 |
|  | CD8+ T Cell | 0.221501 | 0.110943 |
|  | CD4+ T Cell | -0.1748 | 0.210622 |
|  | Macrophage | 0.209332 | 0.132492 |
|  | Neutrophil | 0.308023 | 0.024843 |
|  | Dendritic Cell | 0.103064 | 0.462713 |
| UVM | Purity | -0.26002 | 0.021501 |
|  | B Cell | -0.00916 | 0.937845 |
|  | CD8+ T Cell | -0.37049 | 0.000911 |
|  | CD4+ T Cell | -0.00269 | 0.981584 |
|  | Macrophage | -0.18939 | 0.133912 |
|  | Neutrophil | 0.267462 | 0.018693 |
|  | Dendritic Cell | 0.174915 | 0.136073 |
